# Supplementary material for: Chromothripsis during telomere crisis is independent of NHEJ, and consistent with a replicative origin
Source: Genome Res. 2019 May;29(5):737–49. doi: 10.1101/gr.240705.118 (PMC6499312; doi:10.1101/gr.240705.118)
Supplement: Supplemental Material [file supp_gr.240705.118_Supplemental_file_1.zip › contigs/annotated_contigs/DB80/contig.2.DB80_length_524_mean_cov_8.87404580153.docx]

**DB80_length_524_mean_cov_8.87404580153**

```
T|GGTCTATTTAAAAATGTATACAACTAAATGAATCACTACAACACAATGTGTAAAGTGCATCCATTACATATTTACCAAATGTTACGA
  >chr8:133315574-133315781 + E=6e-110 p=1e-02
CATTCACAACTATGGTTGGGTATTATGGAGGCTTCATGGAGAAAGGGAGAGTTTGGCTGAATTTTGAAGGATGAGTAGGAATTTGGCCA

AGAGGCTGAGAAGAAGGATGTCTAGGCAGAG|AG|AATACTGCATATTCTCTCTTGTAAATGGGAAATAAACCTGTATACATGGACACA
                                  >chr8:133316307-133316621 + E=2e-174
GAGAATGGAATAATAGACAGTGGAGACTCTGAAAGGTGGGAGGGTGGAAGGGAGGTGAAGAATGAGAAATCACCTATTGGGTACGATGT

ACACTATTGGGGTGATGGTTACATAAAAGTCTAGAATTCACCACTATGTAATACATCCATGTAATGAAACTGTACTTGTACCCCCTAAA

TCTACAGAAATGTTTAAAAATGCCATAAAATAAAATGTGGTATAGCCTTAAAAAAATGAAGCTGCACACGTTTGGGGCCAGT
```
